# Supplementary material for: “Stay committed on the frontlines”: sustainability of the activism of social workers in Guiyang, China
Source: PLoS One. 2025 Jun 2;20(5):e0324828. doi: 10.1371/journal.pone.0324828 (PMC12129209; doi:10.1371/journal.pone.0324828)
Supplement: Attachment — Submitted filename: Response to Reviewers.docx [file pone.0324828.s002.docx]

March 8^th^, 2025

Dear reviewers,

We are pleased to resubmit the revised manuscript titled "‘Stay committed on the frontlines’: sustainability of the activism of social workers in Guiyang, China" for your review. We appreciate the time and effort invested by you in evaluating my initial submission and providing valuable feedback to enhance the quality of this study.

We have carefully addressed each of the comments and suggestions, as outlined below. We believe that these revisions have significantly strengthened this manuscript and improved its clarity, coherence, and scholarly contribution.

Thank you once again for being the reviewers of the present study.

Sincerely,

Yifu Liu

# **Response to Reviewer 1:**

We are grateful for your insightful feedback on our paper and the valuable suggestions you've provided to enhance its quality. Your comments have provided us with insights that will undoubtedly strengthen our manuscript. Your comments remind us that when a new theoretical framework shows discrepancies in its application in a certain place, we need to be sensitive and conduct in-depth discussions. And your point about being highly sensitive to the sociocultural context is also well noted. So, we truly appreciate your careful reading, thoughtful feedback and time, which has significantly strengthened our paper.

**The findings of this study present a significant argument that, within the context of China, the model and key factors proposed by the sustained commitment theory do not align with the country's socio-cultural framework. However, the author does not provide a clear discussion explaining the reasons for this inconsistency. It is recommended that a more explicit analysis be included to clarify this issue.**

We acknowledge the reviewer's valid observation regarding the insufficient elaboration on the specified points, which created an impression of limited depth and structural incompleteness in our initial analysis. To address this gap, in the literature review section, we have reviewed the Cultural Opportunity Structure and employed this approach to conduct necessary discussions later in the article. Our revised analysis suggests that participants' demonstrated collectivist and pragmatist orientations likely constitute adaptive strategies shaped by their embeddedness within localized cultural contexts. This theoretical positioning enables us to highlight actors' agency and adaptive capacity in negotiating situational constraints.

**Furthermore, the discussion lacks comparative analysis with studies from other regions, both those with similar and differing socio-cultural contexts. Incorporating such comparisons would provide valuable insights into how the findings align with or diverge from existing research. Therefore, it is suggested that this aspect be addressed to strengthen the discussion.**

We appreciate the reviewer’s astute observation regarding the lack of comparative engagement with existing studies in our original discussion. In response, we have incorporated a dedicated comparative analysis. Through this process, we identified a conspicuous gap in empirical data delineating regional and sociocultural variations in social work retention patterns across China, with prevailing scholarship predominantly relying on generalized assertions, such as the heightened severity of issues in central/western regions compared to coastal developed areas. Notably, existing studies consistently emphasize macro-level interventions, particularly advocating for improved social worker compensation and systemic barrier resolution. Obviously, with the help of Commitment Continuation Theory, the present study fundamentally diverges from these precedents by centering on actors’ capacity, creativity and agency, providing a perspective substantiated by novel empirical contributions. This approach not only addresses the previously missing dimension of "how frontline social workers persist" but also provides critical micro-level insights to complement the dominant discourse on "mitigating social worker workforce attrition." We gratefully acknowledge the reviewer’s guidance in identifying this lacuna, which has enabled us to strengthen both the theoretical coherence and practical relevance of our discussion.

**Additionally, the author is encouraged to review the language for consistency and accuracy. For instance, the term "prosomal growth" (line 408) should be checked for correctness, and the term "frontline" is inconsistently formatted throughout the text—appearing as both "frontline" and "front line." It is recommended that a consistent format be used throughout the manuscript.**

We appreciate your identification of linguistic imprecisions and lack in meticulousness. We have addressed the spelling errors pointed in line 408 and conducted full-text checking to ensure readability and logical coherence. Additionally, we standardized all instances of "front line" to the compound form "frontline" one by one.

**Response to Reviewer 2:**

**The manuscript, “Stay Committed on the Frontlines”: Sustainability of the Activism of Social Workers in Guiyang, China, is a well-structured qualitative research paper that explores the sustainability of social work activism in China, particularly in Guiyang. It provides valuable insights into the experiences of frontline social workers and challenges existing theories on sustained commitment.**

**The study's main contribution lies in its argument that "creativity" is essential for sustaining activism, yet it also introduces "confidence" as a prerequisite, expanding the theoretical framework of sustained commitment. The paper engages in an in-depth discussion of social workers' experiences, making an important empirical and theoretical contribution to social work research.**

**Strengths**

**1. Relevance and Contribution to Social Work and Activism Studies**

**The study tackles an underexplored issue: the sustainability of social work activism in China. This is especially important in a rapidly evolving social and economic landscape.**

**It offers an innovative extension to the sustained commitment theory by introducing "confidence" as a key prerequisite, adding depth to the field of social work activism.**

**The paper provides a detailed, context-specific analysis of frontline social workers, offering valuable qualitative insights.**

**2. Strong Theoretical Foundation**

**The study engages with the sustained commitment theory while also acknowledging its limitations.**

**It effectively critiques and expands the theory, providing a nuanced perspective on activism persistence in social work.**

**The use of historical and theoretical background on the evolution of social work in China strengthens the paper's argument.**

**3. Methodological Rigor**

**The qualitative approach is well-executed, with 15 in-depth interviews across diverse social work fields.**

**The sample represents a range of specializations (e.g., geriatric social work, environmental social work, anti-drug work), making the findings more robust.**

**Thematic analysis is appropriate for the research question, and the paper provides clear justification for its methodological choices.**

**4. Engaging and Clear Presentation**

**The paper is well-structured, following a logical flow from introduction to discussion.**

**The literature review is thorough and well-integrated into the study’s argument.**

**Findings are clearly presented and linked back to theoretical frameworks.**

**5. Ethical Considerations**

**Ethical approval was obtained, and informed consent was secured from all participants.**

**The study demonstrates a strong commitment to confidentiality and ethical research practices.**

We sincerely appreciate your comprehensive and insightful feedback on our paper submitted to the journal. Your thoughtful comments and suggestions provided valuable guidance for improving the quality of our research.

Your suggestions particularly regarding the applicability and the need for deeper theoretical discussion to strengthen the concluding section were invaluable. We understood the importance of addressing these aspects for accurate representation and enhancing credibility of the findings. We endeavoured to provide a better policy recommendation and elaborated on where the focus areas for future research trajectories. Your acknowledgment of the importance using quantitative data or method was duly noted. Please see details of the changes below.

Once again, we sincerely appreciate your time, thoughtful feedback and encouraging remarks about the study's strengths, which is undoubtedly motivation for our continued exploration in this field.

**Areas for Improvement**

**1. Lack of Quantitative Data or Triangulation**

**While the qualitative approach is appropriate, the study could benefit from some quantitative elements (e.g., a survey to validate key themes found in interviews).**

**Triangulation with additional sources, such as policy documents or statistical reports on social worker retention in China, would strengthen the study’s claims.**

Indeed, the original manuscript did not include specific data on social worker attrition. Following the reviewer's precise feedback, I incorporated relevant information into the background section. This includes the loss of certified individuals who chose not to pursue social work as a profession and the attrition rates of professional social workers reported in local studies. The extent of social worker attrition varies significantly across regions, and the level of attention it receives differs as well. Currently, no report provides nationwide data, but existing research is sufficient to confirm the severity of talent loss. Supplementing the study with available data clearly enhances its systematic insight into China’s social work ecosystem, ultimately strengthening the value of present study. I appreciate the reviewer’s valuable suggestion.

Additionally, integrating quantitative elements or adopting a mixed-methods approach would undoubtedly improve the credibility of the study’s conclusions. To address this, I have reinforced the limitations section, explicitly acknowledging the constraints of qualitative research and the potential benefits of incorporating quantitative components. We are indeed considering expanding on this case study with quantitative method for future work—not only to validate the findings but also to provide a more comprehensive examination of social worker attrition.

**2. Limited Generalizability**

**The research focuses only on social workers in Guiyang, which limits its applicability to other regions in China.**

**Given the economic and social differences across China, a comparative study between urban and rural social workers or between different provinces would improve the robustness of the findings.**

The present case study is inherently limited to the specific context of Guiyang, reflecting clear geographical constraints. As is recognized in qualitative research paradigms, transferability constitutes a crucial theoretical consideration; however, the current scarcity of comparable studies in China significantly impedes systematic cross-case analysis. This methodological shortcoming has been explicitly pointed out in the study's limitations section. Looking forward, it is the implication for future research; our research agenda also includes comparative investigations focusing on urban-rural disparities and regional contrasts between China western cities and coastal developed areas. To enhance contextual transparency, we have supplemented the method section with Guiyang's GDP ranking data, thereby strengthening the rationale for site selection. Ultimately, establishing the applicability of these findings to other Chinese regions remains a great focus for future research endeavours.

**3. Deeper Discussion on Policy Implications**

**The paper discusses social workers’ relationships with the government but does not provide enough concrete recommendations on how policies could support sustained activism.**

**How should policymakers and institutions address the issues of low wages, high turnover, and lack of professional recognition? More specific suggestions would make the paper more impactful.**

I have already specified the policy recommendations in accordance with your instruction. These recommendations are based on the improvement of the economic treatment of social workers (as per the research conducted by other scholars). I provided the recommendations from two aspects, creativity and confidence, in line with the sequence of this study. Indeed, specifying these contents at the end of the article has effectively made the entire paper more grounded in practical considerations.

**4. Minor Language and Structural Issues**

**Some sentences could be clearer, and there are occasional grammatical errors (e.g., "event major life aspects such as marriage" should be "even major life aspects such as marriage").**

We extend particular gratitude for your meticulous attention to textual detail in identifying these oversights. Through systematic verification of linguistic inaccuracies and full-text proofreading, we have implemented comprehensive revisions to eliminate potential ambiguities arising from grammatical or orthographic inconsistencies.

**The discussion could be better structured by explicitly separating theoretical contributions from** **practical implications.**

The structure of this part was indeed confusing originally. Now, the paragraphs on theoretical contributions and the suggestions for reality have been clearly separated into two distinct sections to avoid any ambiguity during reading. Thank you for reminding.

**Final Verdict: Suitable for Publication with Minor Revisions**

**The paper is a valuable contribution to social work activism studies. It is well-researched, theoretically sound, and methodologically rigorous. However, addressing the limitations mentioned above—particularly by incorporating more policy recommendations and ensuring language clarity—would enhance its impact and suitability for publication.**

**Recommended Next Steps**

**Clarify and strengthen the** **discussion section by explicitly stating how the findings impact theory, practice, and policy.**

Based on the valuable feedback from you and another reviewer, we have primarily strengthened the discussion section. This includes adding comparisons with other studies, further discussing the reasons for the discrepancies from the theory, and providing actionable policy recommendations, among other things.

**Consider integrating some quantitative validation (if possible in a revision or future study).**

In accordance with your invaluable suggestions, I have incorporated supplementary quantitative data and formulated plans to undertake mixed-method research. To this end, I have emphasized it in the sections of limitations and implications for future research.

**Ensure** **linguistic accuracy by refining sentence structure and fixing minor grammatical errors.**

My co-authors and I have conducted a comprehensive full-text check for grammar and spelling errors to ensure linguistic accuracy.

**Expand the policy discussion to offer concrete solutions for improving the sustainability of social work activism in China.**

As previously indicated, we have developed more targeted policy recommendations building upon the findings of this investigation.

**Response to Editor:**

**Please include the following items when submitting your revised manuscript:**

**• A rebuttal letter that responds to each point raised by the academic editor and reviewer(s). You should upload this letter as a separate file labeled 'Response to Reviewers'.**

We have responded to each of the reviewers' and editors' comments as per your request and have created a separate file named according to the requirements.

**• A marked-up copy of your manuscript that highlights changes made to the original version. You should upload this as a separate file labeled 'Revised Manuscript with Track Changes'.**

As requested, we have prepared the manuscript with all changes. This file has been uploaded separately and labeled as "Revised Manuscript with Track Changes."

**• An unmarked version of your revised paper without tracked changes. You should upload this as a separate file labeled 'Manuscript'.**

We have prepared a clean version of the revised manuscript as instructed, which has been uploaded as a separate file labeled Manuscript.

**If you would like to make changes to your financial disclosure, please include your updated statement in your cover letter. Guidelines for resubmitting your figure files are available below the reviewer comments at the end of this letter.**

**1. Please ensure that your manuscript meets PLOS ONE's style requirements, including those for file naming. The PLOS ONE style templates can be found at**

[**https://journals.plos.org/plosone/s/file?id=wjVg/PLOSOne_formatting_sample_main_body.pdf**](https://journals.plos.org/plosone/s/file?id=wjVg/PLOSOne_formatting_sample_main_body.pdf)

[**https://journals.plos.org/plosone/s/file?id=ba62/PLOSOne_formatting_sample_title_authors_affiliations.pdf**](https://journals.plos.org/plosone/s/file?id=ba62/PLOSOne_formatting_sample_title_authors_affiliations.pdf)

We have carefully adjusted the format throughout the manuscript according to the template provided by PLOS ONE to ensure that our manuscript fully complies with the journal's guidelines. This is a meticulous task, and if there are any oversights or areas that do not meet the requirements, please kindly point them out to us. We would like to make any necessary adjustments promptly.

**2. We note that you have indicated that there are restrictions to data sharing for this study. For studies involving human research participant data or other sensitive data, we encourage authors to share de-identified or anonymized data. However, when data cannot be publicly shared for ethical reasons, we allow authors to make their data sets available upon request. For information on unacceptable data access restrictions, please see http://journals.plos.org/plosone/s/data-availability#loc-unacceptable-data-access-restrictions.**

**Before we proceed with your manuscript, please address the following prompts:**

**a) If there are ethical or legal restrictions on** **sharing a de-identified data set, please explain them in detail (e.g., data contain potentially identifying or sensitive patient information, data are owned by a third-party organization, etc.) and who has imposed them (e.g., a Research Ethics Committee or Institutional Review Board, etc.). Please also provide contact information for a data access committee, ethics committee, or other institutional body to which data requests may be sent.**

**b) If there are no restrictions, please upload the minimal anonymized data set necessary to replicate your study findings to a stable, public repository and provide us with the relevant URLs, DOIs, or accession numbers. Please see http://www.bmj.com/content/340/bmj.c181.long for guidelines on how to de-identify and prepare clinical data for publication. For a list of recommended repositories, please see https://journals.plos.org/plosone/s/recommended-repositories. You also have the option of uploading the data as Supporting Information files, but we would recommend depositing data directly to a data repository if possible.**

**Please update your Data Availability statement in the submission form accordingly.**

We sincerely appreciate your detailed and thoughtful guidance regarding the journal's data sharing policy. While we fully understand PLOS One's requirements, the participants' explicit requests and ethical considerations prevent us from making the complete raw dataset publicly available. Accordingly, we have implemented a controlled data access mechanism through the corresponding author. Additionally, as instructed, we have designated an institutional body as an alternative contact point for data requests. We explained the data access restrictions and the detailed contact information for data requests in the article submitting system (also please see below).

Please advise if any additional modifications or clarifications are required to ensure full compliance with policies. We deeply value your professional guidance throughout this process.

Due to ethical considerations and participants' explicit concerns regarding potential re-identification risks, the original research data cannot be made publicly available. Despite implementing standard anonymization protocols, interviewees expressed apprehension that their identities might be discernible through narrative details in the dataset, leading to their formal objection to public data sharing. In compliance with institutional review board requirements and research ethics standards, access is restricted to de-identified data subsets.

Researchers or institutions requiring data access for legitimate academic purposes may send requests to the corresponding author Dr. Liu ([bestyifu@outlook.com](mailto:bestyifu@outlook.com)). Alternatively, if Dr. Liu is unavailable, inquiries may be directed to Prof. Hu (54375559@qq.com, +86 151 8080 0912), a designated member of the research ethics review committee, school of sociology, Guizhou Minzu University. Please do not hesitate to contact us.

**3. In this instance it seems there may be acceptable restrictions in place that prevent the public sharing of your minimal data. However, in line with our goal of ensuring long-term data availability to all interested researchers, PLOS’ Data Policy states that authors cannot be the sole named individuals responsible for ensuring data access (http://journals.plos.org/plosone/s/data-availability#loc-acceptable-data-sharing-methods).**

**Data requests to a non-author institutional point of contact, such as a data access or ethics committee, helps guarantee long term stability and availability of data. Providing interested researchers with a durable point of contact ensures data will be accessible even if an author changes email addresses, institutions, or becomes unavailable to answer requests.**

**Before we proceed with your manuscript, please also provide non-author contact information (phone/email/hyperlink) for a data access committee, ethics committee, or other institutional body to which data requests may be sent. If no institutional body is available to respond to requests for your minimal data, please consider if there any institutional representatives who did not collaborate in the study, and are not listed as authors on the manuscript, who would be able to hold the data and respond to external requests for data access? If so, please provide their contact information (i.e., email address). Please also provide details on how you will ensure persistent or long-term data storage and availability.**

As outlined above, we have provided a non-author institutional contact point for data access requests through Prof. Hu, a member of the Research Ethics Review Committee at the first author's affiliated university. We believe this arrangement ensures continued data accessibility while maintaining strict adherence to ethical protocols.

**4. Please review your reference list to ensure that it is complete and correct. If you have cited papers that have been retracted, please include the rationale for doing so in the manuscript text, or remove these references and replace them with relevant current references. Any changes to the reference list should be mentioned in the rebuttal letter that accompanies your revised manuscript. If you need to cite a retracted article, indicate the article’s retracted status in the References list and also include a citation and full reference for the retraction notice.**

We have reviewed the reference list to ensure its accuracy and completeness. So far, we have confirmed that none of the cited articles have been retracted. This is the first time we try to submit our paper to PLOS One. While we have implemented cross-checking procedures, we acknowledge the possibility of inadvertent oversights. Should any issues be identified during the editorial review process, we will promptly address them and provide necessary revisions in accordance with journal guidelines.
